# A Multicriteria Decision Making Approach for Estimating the Number of Clusters in a Data Set

**DOI:** 10.1371/journal.pone.0041713

**Published:** 2012-07-27

**Authors:** Yi Peng, Yong Zhang, Gang Kou, Yong Shi

**Affiliations:** 1 School of Management and Economics, University of Electronic Science and Technology of China, Chengdu, Sichuan, China; 2 CAS Research Center on Fictitious Economy and Data Sciences, Beijing, China; 3 College of Information Science & Technology, University of Nebraska at Omaha, Omaha, Nebraska, United States of America; Queen’s University Belfast, United Kingdom

## Abstract

Determining the number of clusters in a data set is an essential yet difficult step in cluster analysis. Since this task involves more than one criterion, it can be modeled as a multiple criteria decision making (MCDM) problem. This paper proposes a multiple criteria decision making (MCDM)-based approach to estimate the number of clusters for a given data set. In this approach, MCDM methods consider different numbers of clusters as alternatives and the outputs of any clustering algorithm on validity measures as criteria. The proposed method is examined by an experimental study using three MCDM methods, the well-known clustering algorithm–*k*-means, ten relative measures, and fifteen public-domain UCI machine learning data sets. The results show that MCDM methods work fairly well in estimating the number of clusters in the data and outperform the ten relative measures considered in the study.

## Introduction

Cluster analysis, the most widely adopted unsupervised learning process, organizes data objects into groups that have high intra-group similarities and inter-group dissimilarities without a priori information. Unlike the evaluation of supervised classifiers, which can be conducted using well-accepted objective measures and procedures, assessment of clustering algorithms’ outputs, often called cluster validation, is challenging because of the lack of objective validation criteria and application-dependent nature of clustering. Nevertheless, cluster validation is necessary to ensure that the resulting clustering structures are not occurred by chance [1].

As an essential step in cluster analysis, cluster validation has been an active research area. Two fundamental issues that need to be addressed in cluster validation are: to estimate the number of clusters in a data set; and to evaluate clustering algorithms [2]. This paper focuses on the first problem. Researchers from several disciplines, such as statistics, pattern recognition, and information retrieval, have studied this issue for years. Marriott (1971) used a heuristic argument to determine the number of clusters in a data set [3]. Hartigan (1975) suggested the statistic *H*(*k*) to estimate the number of clusters [4]. Milligan and Cooper (1985) evaluated thirty procedures for determining the number of clusters using artificial data sets with distinct non-overlapping clusters [5]. The procedures, also called stopping rules, were clustering-algorithm independent and selected from the clustering literature to represent a wide variety of techniques and approaches. Krzanowski and Lai (1988) derived a criterion for determining the number of groups in a data set using sum-of-squares clustering and illustrated that the new criterion has better performance than the Marriott’s criterion [6]. Kaufman and Rousseeuw (1990) used the silhouette statistic to estimate the optimal number of clusters in a data set [7]. Tibshirani et al. (2001) proposed the gap statistic for estimating the number of clusters in a data set and compared the gap method with four other methods in a simulation study [8]. Dudoit and Fridlyand (2002) estimated the number of clusters using a prediction-based resampling method, Clest, and compared the performance of the Clest method with some existing methods using simulated data and gene-expression data [9]. Sugar and James (2003) developed an information theoretic approach for choosing the number of clusters; conducted a simulation study to compare the performance of the proposal with five other methods; and provided a theoretical justification for the proposed procedure [10]. Salvador and Chan (2004) designed the L method to determine the number of clusters for hierarchical clustering algorithms [11].

Different from previously developed approaches, this study examines the problem from a new perspective. Since the determination of the number of clusters in a data set normally involves more than one criterion, it can be modeled as a multiple criteria decision making (MCDM) problem [12], [13]. The objective of this paper is to develop a MCDM-based approach to choose the appropriate number of clusters for a data set. MCDM methods treat different numbers of clusters for a data set as available alternatives and performances of clustering algorithms on validity measures with different numbers of clusters as criteria. Alternatives are then ranked according to the evaluation of multiple criteria. An experimental study is designed to examine the proposed approach using three MCDM methods (i.e., PROMETHEE II, WSM, and TOPSIS), the well-known clustering algorithm–*k*-means, ten relative measures, and fifteen public-domain UCI machine learning data sets. Furthermore, the experimental study applies the ten existing relative measures for estimating the number of clusters and compares their performances with the proposed three MCDM methods.

The rest of the paper is organized as follows. The next section describes the proposed method, the selected MCDM methods, the clustering algorithm, and the validity measures. Results and discussion section presents details of the experimental study and analyzes the results. The last section concludes the paper with summaries, limitations, and future research directions.

## Methods

### Proposed Approach

Estimating the number of clusters for a given data set is closely related to the validity measures and the data set structures. Many validity measures have been proposed and can be classified into three categories: external, internal, and relative [1]. External measures use predefined class labels to examine the clustering results. Because external validation uses the true class labels in the comparison, it is an objective indicator of the true error rate of a clustering algorithm. Internal measures evaluate clustering algorithms by measuring intra- and inter-cluster similarity. An algorithm is regarded as good if the resulting clusters have high intra-class similarities and low inter-class similarities. Relative measures try to find the best clustering structure generated by a clustering algorithm using different parameter values. Extensive reviews of cluster validation techniques can be found in [1] and [14], [15].

**Figure 1:**
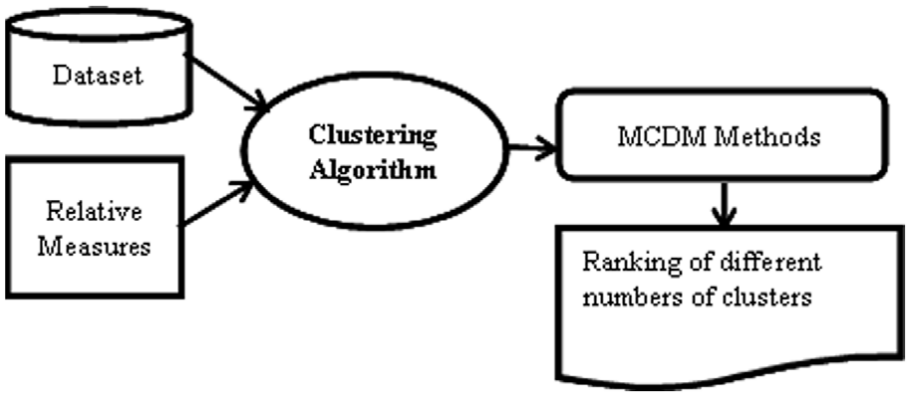
A MCDM-based approach for determining the number of clusters in a dataset.

Although external measures perform well in predicting the clustering error in previous studies, they require a priori structure of a data set and can only be applied to data sets with class labels. Since this study concentrates on data sets without class labels, it utilizes relative validity measures. The proposed approach can be applied to a wide variety of clustering algorithms. For simplicity, this study chooses the well-known *k*-means clustering algorithm. Figure 1 describes the MCDM-based approach for determining the number of clusters in a data set. For a given data set, different numbers of clusters are considered as *alternatives* and the performances of *k*-means clustering algorithm on the relative measures with different numbers of clusters represent *criteria* by MCDM methods. The output is a ranking of numbers of clusters, which evaluates the appropriateness of different numbers of clusters for a given data set based on their overall performances for multiple criteria (i.e., selected relative measures).

### MCDM Methods

This study chooses three MCDM methods for estimating the number of clusters for a data set. This section introduces the selected MCDM methods (i.e., WSM, PROMETHEE, and TOPSIS) and explains how they are used to estimate the optimal number of clusters for a given data set.

### MCDM Method 1: Weighted Sum Method (WSM)

The weighted sum method (WSM) was introduced by Zadeh [16]. It is the most straightforward and widely-used MCDM method for evaluating alternatives. When an MCDM problem involves both benefit and cost criteria, two approaches can be used to deal with conflicting criteria. One is the benefit to cost ration and the other is the benefit minus cost [17]. For the estimation of optimal number of clusters for a data set, the relative indices Dunn, silhouette, and PBM are benefit criteria and have to be maximized, while Hubert, normalized Hubert, Davies-Bouldin index, SD, S_Dbw, CS, and C-index are cost criteria and have to be minimized. This study chooses the benefit minus cost approach and applies the following formulations to rank different numbers of clusters.

Suppose there are *m* alternatives, *k* benefit criteria, and *n* cost criteria. The total benefit of alternative 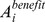 is defined as follows:


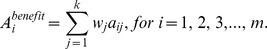


where 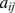 represents the performance measure of the *j*th criterion for alternative 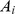. Similarly, the total cost of alternative 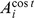 is defined as follows:


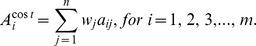


where 
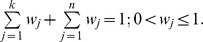
 Then the importance of alternative 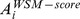 is defined as follows:


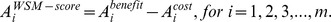


The best alternative is the one has the largest WSM score [18].

### MCDM Method 2: Preference Ranking Organisation Method for Enrichment of Evaluations (PROMETHEE)

Brans proposed the PROMETHEE I and PROMETHEE II, which use pairwise comparisons and outranking relationships to choose the best alternative [19]. The final selection is based on the positive and negative preference flows of each alternative. The positive preference flow indicates how an alternative is outranking all the other alternatives and the negative preference flow indicates how an alternative is outranked by all the other alternatives [20]. While PROMETHEE I obtains partial ranking because it does not compare conflicting actions [21], PROMETHEE II ranks alternatives according to the net flow which equals to the balance of the positive and the negative preference flows. An alternative with a higher net flow is better [20]. Since the goal of this study is to provide a complete ranking of different numbers of clusters, PROMETHEE II is utilized. The following procedure presented by Brans and Mareschal [20] is used in the experimental study:

**Step 1.** define aggregated preference indices.Let *a*, *b*∈*A*, and let : 


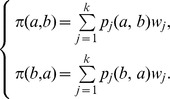


where *A* is a finite set of possible alternatives {a_1_, a_2_,…, a_n_}, *k* represents the number of evaluation criteria, and *w_j_* is the weight of each criterion. For estimating the number of clusters for a given data set, the alternatives are different numbers of clusters and the criteria are relative indices. Arbitrary numbers for the weights can be assigned by decision-makers. The weights are then normalized to ensure that 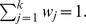 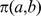 indicates how *a* is preferred to *b* over all the criteria and 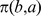 indicates how *b* is preferred to *a* over all the criteria. 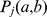 and 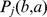 are the preference functions for alternatives *a* and *b*. The relative indices Dunn, silhouette, and PBM have to be maximized, and Hubert, normalized Hubert, DB, SD, S_Dbw, CS, and C-index have to be minimized.

**Step 2.** calculate 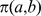 and 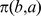 for each pair of alternatives of *A*. There are six types of preference functions and the decision-maker needs to choose one type of the preference functions for each criterion and the values of the corresponding parameters [22]. The usual preference function, which requires no input parameter, is used for all criteria in the experiment.

**Step 3.** define the positive and the negative outranking flow as follows:The positive outranking flow :


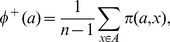


The negative outranking flow :


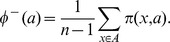


**Step 4.** compute the net outranking flow for each alternative as follows:


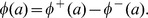


When 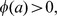 *a* is more outranking all the alternatives on all the evaluation criteria. When 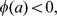 *a* is more outranked.

### MCDM Method 3: Technique for Order Preference by Similarity to Ideal Solution (TOPSIS)

The Technique for order preference by similarity to ideal solution (TOPSIS) method was proposed by Hwang and Yoon [23] to rank alternatives over multiple criteria. It finds the best alternatives by minimizing the distance to the ideal solution and maximizing the distance to the nadir or negative-ideal solution [24]. This paper uses the following TOPSIS procedure, which was adopted from [25] and [24], in the empirical study:

**Step 1.** calculate the normalized decision matrix. The normalized value *r_ij_* is calculated as:


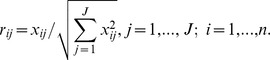


where *J* and *n* denote the number of alternatives and the number of criteria, respectively. For alternative *A_j_*, the performance measure of the *i*th criterion *C_i_* is represented by *x_ij_*.

**Step 2.** develop a set of weights *w_i_* for each criterion and calculate the weighted normalized decision matrix. The weighted normalized value *v_ij_* is calculated as:


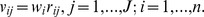


weight of the *i*th criterion, and 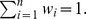


**Step 3.** find the ideal alternative solution *S^+^*, which is calculated as:


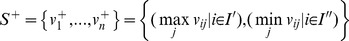


where 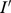 is associated with benefit criteria and 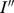 is associated with cost criteria. In this study, benefit and cost criteria of TOPSIS are defined the same as the benefit and cost criteria in WSM.

**Step 4.** find the negative-ideal alternative solution *S^−^*, which is calculated as:


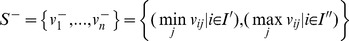


**Step 5.** Calculate the separation measures, using the *n*-dimensional Euclidean distance. The separation of each alternative from the ideal solution is calculated as:


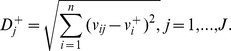


The separation of each alternative from the negative-ideal solution is calculated as:


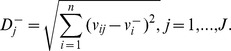


**Step 6.** Calculate a ratio 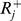 that measures the relative closeness to the ideal solution and is calculated as:


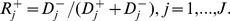


**Step 7.** Rank alternatives by maximizing the ratio 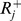.

### Clustering Algorithm

The *k*-means algorithm, the most well-known partitioning method, is an iterative distance-based technique [26]. The input parameter *k* predefines the number of clusters. First, *k* objects are randomly chosen to be the centers of these clusters. All objects are then partitioned into *k* clusters based on the minimum squared-error criterion, which measures the distance between an object and the cluster center. The new mean of each cluster is calculated and the whole process iterates until the cluster centers remain the same [27], [28]. Let 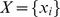, 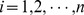 be the *n* objects to be clustered, 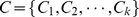 is the set of clusters. Let 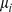 be the mean of cluster 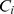. The squared-error between 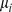 and the objects in cluster 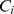 is defined as.


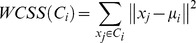


Then the aim of *k*-means algorithm is to minimize the sum of the squared error over all *k* clusters, that is


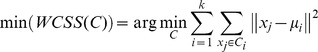


where *WCSS* denotes the sum of the squared error in the inner-cluster.

Two critical steps of *k*-means algorithm have impact on the sum of squared error. First, generate a new partition by assigning each observed point to its closest cluster center, the formula is as follows:


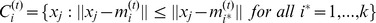


where 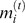 denotes the mean of the 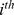 cluster in 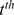
times clustering, while 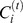 represents all sets contained in the 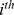 cluster in 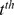 times clustering. Second, compute new cluster mean centers using the following formula.


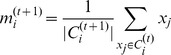


where 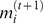 denotes the mean of the 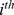 cluster in 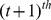 times clustering while 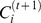 represents all sets contained in the 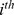 cluster in 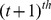 times clustering. The algorithm is implemented using WEKA (Waikato Environment for Knowledge Analysis), a free machine learning software [29].

### Clustering Validity Measures

Ten relative measures are selected for the experiment, namely, the Hubert Γ statistic, the normalized Hubert Γ, the Dunn’s index, the Davies-Bouldin index, the CS measure, the SD index, the S_Dbw index, the silhouette index, PBM, and the C-index. Relative measures can also be used to identify the optimal number of clusters in a data set and some of them, such as the C-index and silhouette, have exhibited good performance in previous studies [5], [8]. The following paragraphs define these relative measures.

- Hubert Γ statistic [30]:

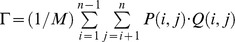


where *n* is the number of objects in a data set, 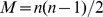, *P* is the proximity matrix of the data set, and *Q* is an 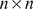 matrix whose (*i*, *j*) element is equal to the distance between the representative points (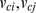) of the clusters where the objects 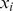 and 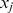 belong [15]. Γ indicates the agreement between *P* and *Q*.

- Normalized Hubert Γ:

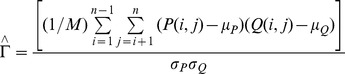


Where 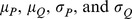 represent the respective means and variances of P and Q matrices [14].

- Dunn’s index [31] evaluates the quality of clusters by measuring inter cluster distance and intra cluster diameter.

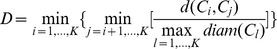


where *K* is the number of clusters, 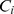 is the *i* ^th^ cluster, 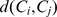 is the distance between cluster 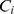 and 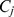, and 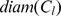 is the diameter of the *l*th cluster. Larger values of *D* suggest good clusters, and a *D* larger than 1 indicates compact separated clusters.

- Davies-Bouldin index is defined as [32]:

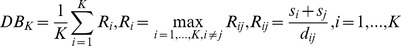


where *K* is the number of clusters, 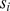 and 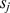 represent the respective dispersion of clusters *i* and *j*, 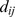 measures the dissimilarity between two clusters, and 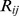 measures the similarity between two clusters [15]. It is the average similarity between each cluster and its most similar one [30].

- The CS measure is proposed to evaluate clusters with different densities and/or sizes [33]. It is computed as:

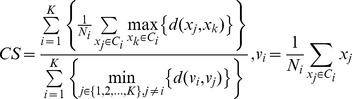


Where 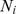 is the number of objects in cluster *i* and *d* is a distance function. The smallest CS measure indicates a valid optimal clustering.

- SD index combines the measurements of average scattering for clusters and total separation between clusters [15]:

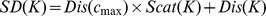


where 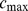 is the maximum number of input clusters, 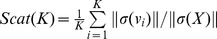, and 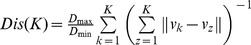, 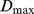 is the maximum distance between cluster centers and the 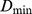 is the minimum distance between cluster centers.

- S_Dbw index is similar to SD index and is defined as [15]:

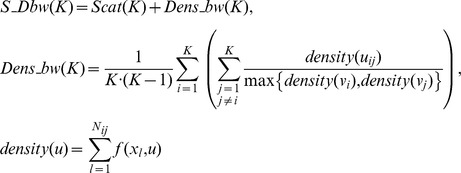


where *N_ij_* is the number of objects that belong to the cluster *C_i_* and *C_j_*, and function *f*(*x,u*) is defined as:


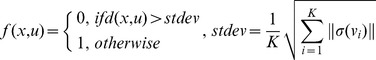

- Silhouette is an internal graphic display for clustering methods evaluation. It represents each cluster by a silhouette, which shows how well objects lie within their clusters. It is defined as [34]:

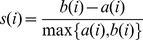


where *i* represents any object in the data set, *a*(*i*) is the average dissimilarity of *i* to all other objects in the same cluster *A*, and *b*(*i*) is the average dissimilarity of *i* to all objects in the neighboring cluster *B*, which is defined as the cluster that has the smallest average dissimilarity of *i* to all objects in it. Note that 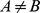 and the dissimilarity is computed using distance measures. Since *a*(*i*) measures how dissimilar *i* is to its own cluster and *b*(*i*) measures how dissimilar *i* is to its neighboring cluster, an *s*(*i*) close to one indicates a good clustering method. The average *s*(*i*) of the whole data set measures the quality of clusters.

- PBM is developed by Pakhira, Bandyopadhyay, and Maulik [35] and it is based on the intra-cluster and inter-cluster distances:

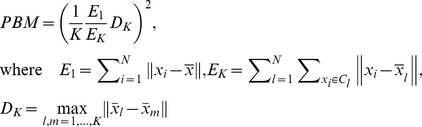

- The C-index [36] is based on intra-cluster distances and their maximum and minimum possible values [37]:

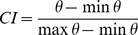
, 
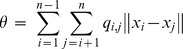


where *q_i,j_*  = 1 if the *i* ^th^ and *j* ^th^ objects are in the same cluster and *q_i,j_*  = 0 otherwise. Small C-index indicates good partitions.

## Results and Discussion

The experiment is designed to examine the proposed MCDM-based approach for estimating the number of clusters in a data set. The data sets, the experimental design, and the results are discussed in sequence.

### Data Sets

Fifteen data sets are used in the experiment. They are provided by UCI machine learning repository (http://archive.ics.uci.edu/ml/) [38]. Table 1 summarizes the characteristics of the data sets.

**Table 1:** Data set structures.

Data Sets	Number of Records	Number of Attributes	Number of Classes
Breast cancer	699	10	2
Breast tissue	106	10	6
Acute inflammations	120	6	2
Ecoli	336	8	8
Glass	214	10	6
Haberman’s survival	306	3	2
Ionosphere	351	34	2
Iris	150	4	3
Parkinsons	197	23	2
Pima Indians diabetes	768	8	2
Sonar	208	60	2
Transfusion	748	5	2
Wine	178	13	3
Wine quality (red)	1599	11	6
Yeast	1484	8	10

**Table 2:** Rankings of numbers of clusters for the yeast data set.

	PROMETHEE II		TOPSIS		WSM	
Number ofclusters	Value	Order	Value	Order	Value	Order
K = 2	−0.2265	8	0.400601	9	−0.25409	9
K = 3	0.1125	3	0.537494	5	−0.1994	3
K = 4	−0.17975	7	0.451931	8	−0.2342	7
K = 5	0.102	4	0.539354	4	−0.2154	4
K = 6	−0.31675	9	0.481188	7	−0.2463	8
K = 7	0.02575	5	0.544836	3	−0.2213	5
K = 8	−0.10825	6	0.529223	6	−0.2336	6
K = 9	0.29475	2	***0.626924***	***1***	***−0.1827***	***1***
***K = 10***	***0.29625***	***1***	0.603641	2	−0.185	2

The breast cancer data set was provided by Dr. William H. Wolberg from the University of Wisconsin Hospitals [39]. Each record has ten attributes to describe cytological characteristics of breast and belongs to either benign or malignant class. The breast tissue data set contains impedance measurements of freshly excised tissue samples from the breast [40]. The acute inflammations data set includes examples of diagnosing of the acute inflammations of urinary bladder and acute nephritises [41]. The ecoli data set contains protein localization sites [42]. The glass data set describes six types of glass in terms of their oxide content [43]. The Haberman’s survival data set includes samples from a study that was conducted between 1958 and 1970 on the survival of patients who had undergone surgery for breast cancer [44]. The Ionosphere data set describes radar data return from the ionosphere [45]. The iris data uses length and width of sepal and petal to describe three types of iris plant [46]. The Parkinson’s data set consists of a range of biomedical voice measurements from people who are either healthy or with Parkinson’s disease [47]. The Pima Indians diabetes data set uses several aspects to separate females from Pima Indian heritage who are either healthy or with diabetes [48]. The sonar data set collects data obtained by bouncing sonar signals off a metal cylinder and rocks at various angles and under various conditions [49]. The transfusion data set has four aspects of blood donors, i.e., months since last donation, total number of donation, total blood donated, and months since first donation [50]. The wine data uses constituents found in wines to distinguish three types of wine [51]. The wine quality (red) data set contains inputs from physicochemical tests to describe red variant of the Portuguese “Vihno Verde” wine [52]. The yeast data set collects the amino acid sequence information to predict the cellular localization sites of proteins [53].

### Experimental Design

The experiment is designed for two purposes: (1) examine the effectiveness of the proposed approach and (2) compare the proposed approach with existing methods. The effectiveness of the proposed approach is examined by applying three MCDM methods to estimate the number of clusters for fifteen public-domain UCI machine learning data sets. The performances of the three MCDM methods are then compared to the ten relative measures presented in the previous section using the same sets of UCI data [54].

The experiment is carried out according to the following process:

**Input.** fifteen UCI machine learning data sets.

**Output.** Rankings of different numbers of clusters for each data set by the MCDM methods and the relative measures.

**Step 1.** Prepare the data sets: remove class labels from the data sets and upload the data sets to Weka 3.6.

**Step 2.** Get clustering solutions using the *k*-means algorithm for all data sets.

**Step 3.** For each data set, the *k*-means algorithm is used to compute the ten selected relative measures nine times, each time with a different number of clusters (i.e., from 2 to 10).

**Step 4.** For each data set, generate the optimal number of clusters determined by each relative measure.

**Step 5.** Twelve domain experts were asked to assign weights to relative measures for each data set based on their experiences. The score ranges from 0 to 10 with increasing importance, and the averaged and normalized scores are weights of relative measures.

**Step 6.** Generate three rankings of different numbers of clusters using PROMETHEE II, WSM, and TOPSIS for the data sets. For each data set, different numbers of clusters are alternatives and the performances of *k*-means algorithm on the relative measures are criteria. PROMETHEE II was implemented by the MCDM software D-Sight, and WSM and TOPSIS were implemented using MATLAB 7.0 [54]. If the top-three ranked numbers of clusters have very close ranking values (i.e., the difference between their values is less than 0.01), both the ranking order and ranking values should be provided to the decision-maker.

**Table 3:** Estimations of number of clusters by the relative measures.

	Relative measures										
Data sets	Dunn	Sil	PBM	Hubert	Normalized Hubert	DB	SD	S_Dbw	CS	C-index	#Cluster
Breast cancer	5	***2***	***2***	***2***	***2***	10	***2***	10	***2***	5	2
Breast tissue	3	2	***6***	2	2	3	2	7	***6***	10	6
Acute inflammations	4	***2***	9	***2***	***2***	10	4	10	9	4	2
Ecoli	3	2	3	2	2	10	4	7	4	4	8
Glass	2	2	2	2	2	2	2	10	8	2	6
Haberman’s survival	8	***2***	5	***2***	***2***	10	4	10	4	10	2
Ionosphere	***2***	***2***	***2***	***2***	3	10	***2***	9	9	10	2
Iris	2	2	2	2	2	2	2	10	2	2	3
Parkinsons	3	3	5	***2***	***2***	8	3	9	8	10	2
Pima Indians diabetes	***2***	***2***	4	***2***	***2***	10	3	10	10	10	2
Sonar	4	***2***	***2***	***2***	***2***	10	4	10	4	4	2
Transfusion	9/10	***2***	7	***2***	***2***	***2***	***2***	10	7	9	2
Wine	6	***3***	***3***	2	2	***3***	2	7	***3***	6	3
Wine quality (red)	2	2	3	2	2	9	3	3	3	9	6
Yeast	***9/10***	2	2	2	2	***10***	3	9	***10***	***10***	10

**Table 4:** Estimations of number of clusters by the MCDM methods.

	MCDM Methods			
Data sets	PROMETHEE II	TOPSIS	WSM	#Cluster
Breast cancer	***2***	***2***	***2***	2
Breast tissue	***6***	***6***	***6***	6
Acute inflammations	***2***	4	4	2
Ecoli	4	3	3	8
Glass	8	2	2	6
Haberman’s survival	***2***	***2***	***2***	2
Ionosphere	***2***	***2***	***2***	2
Iris	2	2	2	3
Parkinsons	5	3	3	2
Pima Indians diabetes	***2***	***2***	***2***	2
Sonar	***2***	***2***	***2***	2
Transfusion	***2***	***2***	***2***	2
Wine	***3***	***3***	***3***	3
Wine quality (red)	***6***	***6***	3	6
Yeast	***10***	9	9	10

**Table 5:** Results summary.

	Relative Measures										MCDM Methods		
	Dunn	Silhouette	PBM	Hubert	Normalized Hubert	DB	SD	S_Dbw	CS	C-index	PROMETHEE	TOPSIS	WSM
Correct number	3	8	5	8	7	3	3	0	4	1	11	9	8

**END**

For each data set, nine different numbers of clusters (i.e., from 2 to 10) are used as alternatives in the MCDM methods due to the structures of these data sets (refer to Table 1). When the structure of a data set is unknown, reasonable numbers of clusters can be used as alternatives.

The 0–10 scale used by domain experts indicates increasing importance of criteria. Number 0 indicates that the domain expert is not interested in that criterion and number 10 indicates that the domain expert considers the criterion extremely important. Number 5, the midpoint of the scale, shows the moderate importance of a criterion. Domain experts can use numbers 1, 2, 3, and 4 to represent the importance between none and moderate, with increasing strength. Similarly, numbers 6, 7, 8, and 9 are used to represent the importance between moderate and extreme, with increasing intensity. Since the weights of criteria have important impact on the final evaluation of alternatives, some MCDM softwares provide tools to facilitate sensitivity and robustness analyses. For instance, the D-Sight software allows the decision-maker to find out the stability intervals of the weights of criteria and observe the impact of a change of weight on the final ranking.

### Experimental Results and Discussion

To illustrate the values and rankings generated by the MCDM methods for different numbers of clusters [55], Table 2 presents the yeast data set as an example. The number of classes provided by UCI machine learning repository for yeast is ten. As can be seen from Table 2, PROMETHEE II finds the right number of clusters for this data set. Both TOPSIS and WSM rank *K* = 9 as the best alternative and *K* = 10 as the second best.

Table 3 and Table 4 summarize the best ranked numbers of clusters for all data sets produced by the ten relative measures and the three MCDM methods, respectively. Both tables have the same structure. The leftmost column lists the data sets and the rightmost column gives the number of classes provided by UCI machine learning repository for each data set. The entries in the middle of Table 3 and 4 show the optimal number of clusters for each data set determined by the relative measures and the MCDM methods, respectively. The correctly estimated numbers of clusters are highlighted in boldface and italic. Table 5 summarizes the number of correct determinations for the three MCDM methods and the ten relative measures.

A number of observations can be made based on the experimental study. First, the proposed approach is effective at estimating the optimal number of clusters in data. WSM, TOPSIS, and PROMETHEE II can estimate the optimal numbers of clusters for eight, nine, and eleven datasets, respectively. Second, the three MCDM methods outperform the ten existing relative measures considered in this study. The best performance of the relative measures (i.e., Silhouette and Hubert) is equal to the worst performance of the three MCDM methods (i.e., WSM). Furthermore, as can be seen from Table 3 and 4, the data sets that were missed by the MCDM methods were also missed by the relative measures, except the Parkinson’s data set. Third, the estimation of numbers of clusters for a given data set generated by different MCDM methods may vary. Fourth, there are situations that the top-ranked numbers of clusters by MCDM methods have very close ranking values. For instance, 9 and 10 were ranked by WSM as the best and the second best choices for the yeast data set, respectively (Table 2). But the difference between their WSM scores is only 0.0023. In such a case, both 9 and 10 and their corresponding ranking values should be provided to the decision-maker.

### Conclusions

Determining the number of clusters in a data set is intrinsically difficult because this is often a subjective process. This paper has proposed a MCDM-based approach for estimating the optimal number of clusters in a data set, which treats different numbers of clusters as alternatives and clustering validity measures as criteria. Different numbers of clusters are ranked according to the corresponding performances of clustering algorithms on validity measures. The top ranked number of clusters is the one with the best overall performances for all the selected validity measures.

The experiment is designed to examine the effectiveness of the proposed method and compare the new approach with existing methods using three MCDM methods (WSM, TOPSIS, and PROMETHEE II), the *k*-means clustering algorithm, ten relative measures, and fifteen public-domain UCI machine learning data sets. The results prove the effectiveness of the proposed approach in estimating the number of clusters. Specifically, WSM, TOPSIS, and PROMETHEE II can estimate the optimal numbers of clusters for eight, nine, and eleven datasets, respectively. The comparative study shows that the three MCDM methods outperform the ten existing relative measures considered in the present study. The best performance of the relative measures (i.e., Silhouette and Hubert) is equal to the worst performance of the three MCDM methods (i.e., WSM).

MCDM methods normally require decision makers or domain experts to provide weights for the criteria involved in the decision problem. In this study, the proposed approach needs domain experts to assign weights for the relative measures. When automatic decision process is required or inputs of criteria weights from domain experts are unavailable, it is necessary to find a way to obtain the weights automatically and this is a future research direction. In addition, different MCDM methods may generate different rankings of the numbers of clusters. How to reconcile these differences is another future research avenue. This study only considers validity indices for crisp clustering. However, many real-life data sets have overlapping clusters, whose boundaries are hard to define. Therefore a potential direction of future work is to introduce validity indices that are suitable for fuzzy clustering to MCDM methods.
